# Peripheral infusion of rat bone marrow derived endothelial progenitor cells leads to homing in acute lung injury

**DOI:** 10.1186/1465-9921-8-50

**Published:** 2007-07-09

**Authors:** Christian M Kähler, Jutta Wechselberger, Wolfgang Hilbe, Andreas Gschwendtner, Daniela Colleselli, Harald Niederegger, Eva-Maria Boneberg, Gilbert Spizzo, Albrecht Wendel, Eberhard Gunsilius, Josef R Patsch, Jürg Hamacher

**Affiliations:** 1 Department of Internal Medicine, Division of General Internal Medicine, Pneumology Centre, Innsbruck Medical University, Austria; 2 Department of Internal Medicine, Division of General Internal Medicine, Oncology Service, Innsbruck Medical University, Austria; 3 Department of Pathology, Innsbruck Medical University, Austria; 4 Department of Experimental Pathology, Innsbruck Medical University, Austria; 5 Biotechnology Institute Thurgau, University of Konstanz, Tägerwilen, Switzerland; 6 Department of Internal Medicine, Division of Haematology and Oncology, Innsbruck Medical University, Austria; 7 Biochemical Pharmacology, Faculty of Biology, University of Konstanz, Germany; 8 Pulmonary Division, Department of Internal Medicine, University Hospital of Homburg, University of Saarland, D-66421 Homburg, Germany

## Abstract

**Background:**

Bone marrow-derived progenitors for both epithelial and endothelial cells have been observed in the lung. Besides mature endothelial cells (EC) that compose the adult vasculature, endothelial progenitor cells (EPC) are supposed to be released from the bone marrow into the peripheral blood after stimulation by distinct inflammatory injuries. Homing of *ex vivo *generated bone marrow-derived EPC into the injured lung has not been investigated so far. We therefore tested the hypothesis whether homing of EPC in damaged lung tissue occurs after intravenous administration.

**Methods:**

Ex vivo generated, characterized and cultivated rat bone marrow-derived EPC were investigated for proliferation and vasculogenic properties in vitro. EPC were tested for their homing in a left-sided rat lung transplant model mimicking a severe acute lung injury. EPC were transplanted into the host animal by peripheral administration into the femoral vein (10^6 ^cells). Rats were sacrificed 1, 4 or 9 days after lung transplantation and homing of EPC was evaluated by fluorescence microscopy. EPC were tested further for their involvement in vasculogenesis processes occurring in subcutaneously applied Matrigel in transplanted animals.

**Results:**

We demonstrate the integration of intravenously injected EPC into the tissue of the transplanted left lung suffering from acute lung injury. EPC were localized in vessel walls as well as in destructed lung tissue. Virtually no cells were found in the right lung or in other organs. However, few EPC were found in subcutaneous Matrigel in transplanted rats.

**Conclusion:**

Transplanted EPC may play an important role in reestablishing the endothelial integrity in vessels after severe injury or at inflamatory sites and might further contribute to vascular repair or wound healing processes in severely damaged tissue. Therapeutic applications of EPC transplantation may ensue.

## Background

Aimed at a huge surface between blood and ambient air to accomplish the optimal external breathing, the lung is a high-throughput blood spongue that has matched its endothelial surface virtually to the same size as the alveolar space [1]. Endothelial cells (EC) regulate the transport of nutrients and mediators, the traffic of inflammatory cells, and regulate the vascular tone, density and selectivity of the blood-interstitial barrier [2]. In many pathophysiologic processes, e.g. during haemostasis, inflammation and angiogenesis they thus are suggested to play a key role [3].

Due to the lung's serial position in the blood circulation the whole amount of cardiac output has to pass through the pulmonary capillary network, giving the lung an important role as a capillary filter. This capillary network has furthermore been organized as an intravascular storage pool for polymorphonuclear neutrophil granulocytes (PMN). This strategical position in a serially circulated organ like the lung may be an advantage to rapidly overcome infective agents, but may be dangerous in case of overwhelming inflammatory stimuli during pneumonia, trauma or sepsis, conditions that may cause acute lung injury (ALI). ALI and consecutively, the Acute Respiratory Distress Syndrome (ARDS) are characterized by a diffuse transmural alveolar wall damage leading to severe epithelial injury and cell death [4]. Pulmonary EC death and dysfunction of the vessel network seem to be characteristic for this severe lung damage which is still leading in a high proportion to patients death [5]. Besides vascular lesions in main pulmonary arteries [6], up to 50% of lung capillaries have been shown to be lost during ALI/ARDS [7]. The importance of EC cell death has been further supported by data observed in animal models inducing ALI after lipopolysaccharide injection [8,9]. Severe tissue injury in ALI/ARDS is suggested to result further in an acute inflammatory response followed by repair processes that may result in additional apoptosis/necrosis of EC or epithelial cells [3,8-10]. The replacement of these dead cells during this repair process was formerly uniquely believed to be derived from cells in the vicinity of the damage within a given tissue [11]. However, recently published data suggest that repair mechanisms may in part also rely on bone marrow-derived progenitor cells that are capable of differentiating in the directions that the injured site needs [3,12,13], and that there is a dose-relationship beween the degree of lung injury and the amount of repair cells stemming from the bone marrow [14].

Indeed, bone marrow has become a recognized source for progenitor cells of several cell types [15], including EC [13,16,17], epithelial cells [13,18-23], mesenchymal stem cells [24,25], hepatocytes [26], cardiac [27], striated [28] and smooth muscle cells [29], fibroblasts or myofibroblasts [30,31] and neurons [32,33]. However, a number of observations have been made on rare engrafted cells, where circulating blood cells, dead cells, cell fusion, or artifacts like autofluorescence might lead to misinterpretation. Therefore, the reconstitution of lung epithelium by bone marrow cells has recently been questioned [34].

Nevertheless, therapeutic trials aiming for organ repair utilising cell progenitors are evolving [35-42]. Additionally, EPC can circulate in the peripheral blood and track to other organs [17,43].

In contrast to mature EC that compose the adult vasculature, EPC are supposed to be released from the bone marrow into the peripheral blood after stimulation by distinct inflammatory injuries [3,44]. EPC have been shown to display a higher proliferative potential [45] and may migrate to regions of the circulatory system with injured endothelia, including sites of traumatic, degenerative, or ischemic injury and thus promote repair or the formation of new vessels [13,45-52]. Whereas in blood the mature endothelial cells may originate from sloughing off the vessel wall following some form of vascular insult, higher numbers of circulating EPC seem consistently associated with a more normal vascular function or less endothelial dysfunction, and less cardiovascular risk factors [43], cardiovascular events and death [53]. Functional circulating EPC are thus interpreted as the repair cells of vascular beds [54]. A recent study suggests a superior survival of patients with acute lung injury and higher number of circulating EPC than their counterparts with lower numbers [55]. Also in pneumonia patients, circulating EPC increase. Imaging data further imply persistent fibrotic changes if circulating EPC numbers remained low during pneumonia, therefore suggest some role in the evolution or repair of such tissular injury [56]. In healthy adults, the concentration of EPC in peripheral blood is low (2–3 cells/ml) [57], but vastly depends on the determination technique [54]. EPC levels have been shown to be about threefold higher in human umbilical cord blood.

In this study we tested the hypothesis whether the homing of intravenously administered bone marrow-derived EPC occurred in damaged lung tissue after the setting of severe tissue injury, as previously shown in part in an abstract [58]. As these cells are suggested to be important for repairing tissue damage, are rather homogeneous compared to bone marrow [59], and we ought to investigate their presence in the lung, we chose a unilateral model of severe ALI. Due to a prolonged ischemia of 20 h, such severe lung injury occurred as ischemia-reperfusion injury in a model of left-sided rat lung allotransplantation. Such transplantation of EPC would primarily elucidate key pathogenic aspects of repair. It may also open prospects to modulate biological responses by such cells for gene delivery, drug- or chemosensitization or apoptosis in tumor vasculature as Trojan horses [60].

## Methods

### Isolation and culture conditions of endothelial progenitor cells (EPC) from rat bone marrow

EPC were collected from the femurs of 6 to 8 weeks old male Sprague-Dawley rats (220–280 g). Aspirated bone marrow was mixed with 1000 U/ml heparin (Immuno, Vienna, Austria), deoxyribonuclease I 1000 U/ml (Sigma, St. Louis, MO) in Dulbecco's PBS (PAA Laboratories, Austria) as described [61]. The mononuclear cell fraction was obtained from a Lymphoprep density gradient (Nycomed, Norway) after centrifugation for 30 min at 1700 rpm (centrifuge GPR, Beckman, Hettich, Germany). The mononuclear cell fraction was carded, washed and centrifuged at 800 rpm for 10 min. The cell pellet was then suspended in EBM-2 medium (Clonetics, San Diego, California) supplemented with 20% fetal calf serum (FCS, PAA Laboratories, Austria) and plated on rat-derived fibronectin-coated (10 μg/ml, Sigma, F0635, St. Louis, MO) 12-well plates (Costar, Corning, The Netherlands). After 24 h the non-adherent cell population was aspirated and transferred to a new fibronectin-coated plate. After another 24 h this procedure was repeated to remove rapidly adherent hematopoietic cells or mature EC being possibly present in the aspirate. Only the non-adherent cell population harvested after 48 h was evaluated further in all experiments. This fraction was cultured in EBM-2 medium containing vascular endothelial growth factor (VEGF), human fibroblast growth factor-B (hFGF-B), R^3^-insulin like growth factor (R^3^-IGF-1), human epidermal growth factor (hEGF), ascorbic acid, hydrocotisone, gentamycin, amphotericin B (MV-Kit, Clonetics, San Diego, California) and stem cell growth factor (SCGF, PreproTech EC Ltd., USA). After 2–3 days a kind of angioblast-like cells were observed and spindle-shaped cell outgrowth documented. After 7 to 10 days confluence of the outgrowing cell population was reached and cells were divided by collagenase (Type CLS-CI-22, Biochrom AG, Berlin, Germany).

### Characterization of EPC from rat bone marrow

Cells were primarily characterized by phase contrast microscopy evaluating cobblestone morphology which is typical for confluent EC. EPC were further imaged for their incorporation of acetylated low density lipoprotein (aLDL) labeled with fluorescent Dil dye (Dil-acLDL; Biomedical Technologies, Stoughton, Massachusetts). Indirect immunofluorescence for detection of CD31 (PharMingen, USA), was performed using rabbit anti-rat PECAM-1 antibody by a standard protocol as given by the manufacturer. Secondary FITC-labeled antibodies (swine anti-rabbit Ig) were purchased from DAKO (Carpenteria, California). Von Willebrand Factor (vWF) was detected by direct immunofluorescence using a FITC-marked anti-vWF antibody (DAKO, Carpenteria, California). Direct and indirect immunofluorescence microscopy was done using a Olympus BH-2 RFCA fluorescence microscope and KAPPAImage software (Kappa Optoelectronics, Germany).

Additionally, flow cytometry (FACS) analyses were performed for further characterization of EPC. EPC were checked for the presence of CD146-PE (P1H12) (Chemicon, Temecula, USA), CD133-PE (Milteny-Biotec, Bergisch-Gladbach, Germany), VEGF receptor-2 (KDR; R&D, Wiesbaden, Germany) and CD106 (clone 1.G11B1, Serotec, Oxford, UK). Expression of cell surface markers were measured in a LSR flow cytometer (Becton Dickinson, USA) using the Cell Quest software (Becton Dickinson, USA).

### Isolation and culture conditions of arterial endothelial cells from rat thoracic aorta (rAEC)

Female Sprague-Dawley rats weighing 230–280 g were housed in a light-, temperature-, and humidity-controlled environment and provided with food and water ad libitum. Before killing by decapitation, rats were anesthetized with dietylether and thoracic aortas prepared immediately after removal. Aortas were cut into consecutive 2 mm segmental rings, mounted on the plastic surface of 24-well tissue culture plates coated with a distinct mixture of collagen type I (0.1 mg/ml; Collaborative Biomedical Products, Bedford, MA), fibronectin (10 μg/ml; Collaborative Biomedical Products) and porcine gelatin (0.2%; Sigma, St. Louis, MO). Cells were cultured in M199 with 10% FCS, 100 U/ml penicillin, 100 mg/ml streptomycin and 100 mg/ml endothelial cell growth factor supplement (Sigma, St. Louis, MO) and kept in a humidified incubator at 37°C in 5% CO_2_. Rat aortic endothelial cells (rAEC) were used between passages three and five for all experiments.

### Isolation and culture conditions of arterial endothelial cells from rat pulmonary arteries (rPAEC)

As given above two female Sprague-Dawley rats weighing 230–280 g were killed by decapitation: rats were anesthetized with dietylether and main pulmonary arteries prepared immediately after removal. Pulmonary arteries were cut into consecutive 2 mm segmental rings, mounted on the plastic surface of 24-well tissue culture plates coated with rat 10 μg/ml fibronectin. Rat pulmonary artery endothelial cells (rPAEC) were cultured in endothelial culture medium (Promo Cell, Heidelberg, Germany) containing 10% FCS and 2% endothelial cell growth supplement (Promo Cell, Heidelberg, Germany), 1% penicillin/streptomycin solution (Sigma, St. Louis, MO) and kept in a humidified incubator at 37°C in 5% CO_2_. rPAEC were used between passages three and five for all experiments.

### Culture conditions of human lung microvascular endothelial cells (hL-MVEC)

Primary human lung microvascular endothelial cells (hL-MVEC; Clonetics, San Diego, CA, USA) were cultured according to the manufacturer's protocol in EBM-2 medium containing vascular endothelial growth factor (VEGF), human fibroblast growth factor-B (hFGF-B), R^3^-insulin like growth factor (R^3^-IGF-1), human epidermal growth factor (hEGF), ascorbic acid, hydrocotisone, gentamycin, amphotericin B (MV-Kit, Clonetics, San Diego, California).

### Proliferation experiments

After incubation at 37°C for various time periods cellular proliferation was measured using a colorimetric assay for cell growth and chemosensitivity. This colorimetric assay based on the tetrazolium salt MTT ((3-(4,5-dimethyldiazol-2-yl)-2,5-diphenyl tetrazolium bromide; Sigma, St. Louis, MO) detects living but not dead cells, and the signal generated is directly proportional to the number of cells [62]. After 6 h of incubation, medium was aspirated from adherent cells without disturbing formazan crystals formed within the cells. Subsequently, dimethylsulfoxide (Merck, Darmstadt, Germany) was added to each well, the plates were agitated on a plate shaker, and the optical density was read with an enzyme-linked immunoabsorbent assay reader at 570 nm (MR 700; Dynatech Labs, Guernsey, United Kingdom).

### In vitro capillary tube formation assay in Matrigel

For analysis of capillary tube formation, 150 μl Matrigel (Becton Dickinson, Heidelberg, Germany), an extracellular mouse sarcoma matrix (Engelbreth-Holm-Swarm tumor) known to be *in vivo *and *in vitro *a pro-angiogenic stimulus, was laid into the wells of a 48-well plate (Falcon, Heidelberg, Germany) and incubated at 37°C for 60 minutes. EPC or hL-MVEC were harvested and 3 × 10^4 ^cells resuspended in 200 μl EBM-2/MV medium and plated. Conditions with EBM-2/MV with 10% FCS or supplemented with 50 ng/ml VEGF were studied. Capillary tube formation on Matrigel was observed under an inverted Zeiss Axiovert microscope after 5 or 18 h of incubation.

### Application of subcutanous Matrigel

200 μl of Matrigel (Becton Dickinson, Heidelberg, Germany) was subcutanously administered into the left-sided flank subcutis of lung transplant recipients with EPC injection eight days before transplantation in order to assess angiogenesis as shown in figure five.

### Ex vivo cell tracer labeling of EPC

EPC were kept on fibronectin-coated culture flasks within EBM-2/MV medium as given above without further complementation prior to in vivo coloration. After a washing procedure in buffer solution EPC were stained with the anionic sulfophenyl cell tracer SP-DiIC_18_(3) (Molecular Probes, Leyden, The Netherlands), a formaldehyde and acetone resistant Dil dye at a concentration of 2 μg/ml solution in standard PBS. Staining was performed on adherent EPC at 37°C for 10 min followed by a further incubation period of 35 min at 4°C. After staining, cells were washed in EBM-2 supplemented with 10% FCS. Efficiacy of coloration and cell morphology was checked by fluorescence microscopy twice before transplantation. Furthermore, growth, morphology and fluorescence intensity of SP-DiIC_18_(3)-in vivo staining was checked at the end of each experiment. No differences in biological functions of SP-DiIC_18_(3)-stained EPC tested have been observed (data not shown). SP-DiIC_18_(3) staining was detectable up to 14 days in *in vitro *cultured EPC (data not shown).

### Flow cytometry (FACS) of EPC in rat blood samples

100 μl of EDTA blood was withdrawn from an EPC-injected lung transplant recipient 12 h post reperfusion from the jugular vein. Whole blood was stained with 10 μl anti rat CD42d-FITC (Becton Dickinson, Heidelberg, Germany), 10 μl anti rat CD45-FITC (Becton Dickinson), and 10 μl anti human CD146-PE (clone P1H12, Chemicon, Hofheim, Germany) for 30 min at room temperature. Red blood cells were lysed with 1 ml of BD Lysing Solution (Becton Dickinson) for 10 min at room temperature. After washing twice with 3 ml PBS, cells were measured in a BD LSR flow cytometer (Becton Dickinson) using Cell Quest software (Becton Dickinson). To quantify the amount of circulating EC in the blood samples a standardized amount of 6 μm latex microspheres (Polyscienes, Eppelheim, Germany) was added to each blood sample. With this internal standard it was possible to calculate the amount of circulating EC per ml of blood.

### In vivo experimental protocol including the intravenous injection of EPC

All experiments were performed according to the Helsinki convention for the use and care of animals and were approved by the local review boards for animal care. Briefly, weight matched female Sprague-Dawley rats of 220 – 270 g received orthotopic single left lung allografts under general anesthesia with 2% halothane from female Sprague-Dawley rats after a total graft ischemia of 20 h. A standard cuff technique for the vessel anastomoses and a running suture for the bronchial anastomosis were applied, as well as for the donor procedure and transplantation [63]. Immediately before injection of EPC into the host rat, SP-DiIC_18_(3)-labelled cells were harvested, washed and resuspended in EBM-2 medium at a concentration of 1 × 10^6^/ml. Injection of EPC was done under general anesthesia with 2% halothane into the saphenous vein of the right hind leg under microscopic vision to ascertain the successful and complete venous administration into each host animal. Intravenous application of EPC was performed 50 to 120 min after reperfusion of the transplanted left lung (n = 9). Two further control animals were not lung transplanted but received labelled EPC as given above.

### In vivo experimental protocol

#### Host animals

Weight matched female Sprague-Dawley rats of 220 – 270 g received orthotopic single left lung allografts from female Sprague-Dawley rats after a total graft ischemia of 20 h. A cuff technique for the vessel anastomoses and a running suture for the bronchial anastomosis were applied. The experiments were performed according to the Helsinki convention for the use and care of animals and were approved by the local review boards for animal care.

#### Donor procedure

Animals were anaesthetized by intraperitoneally administered pentobarbital (50 mg/kg) and heparinized (500 I.U./kg). After tracheotomy the animals were ventilated through a 14 gauge cannula (FiO_2 _= 1.0) by a Unno rodent ventilator (Hugo Sachs Harvard Apparatus, March-Hugstetten, Germany) at a tidal volume of 8 ml/kg at 100/min. After division of the inferior vena cava and resection of the left appendix of the heart, a small silicon tube was inserted into the main pulmonary artery. Both lungs were flushed with 20 ml of Low Potassium Dextrane (LPD) solution (Perfadex, kindly provided from Xvivo, Göteborg, Sweden) at a pressure of 20 cm H_2_O. The trachea was tied in end-inspiration, the heart-lung block removed and 16 gauge cuffs (Abbocath-T, Abbott, Sligo, Ireland) were placed around the pulmonary artery and vein. The vessels were inverted and tied onto the cuff with an 8-0 monomeric filament. The lung was stored in LPD solution at 1.5°C until implantation.

#### Recipient procedure

Transplantation was performed after 20 h of cold ischemia at 1.5°C. The recipient rat was anesthetized by breathing 4% halothane in a glass chamber followed by intubation. Anesthesia was maintained throughout the operative procedure with 2% halothane. A left lateral thoracotomy was performed in the 4^th ^intercostal space. The left hilum was dissected and after clamping of the left pulmonary artery and vein with removable microvascular clips, the pulmonary vein was opened, flushed with heparinized saline solution, and the cuff was inserted and fixed with 6-0 Silk. With the same technique, the pulmonary artery was anastomosed. The native left lung was removed and the bronchial anastomosis performed with a running over-and-over suture with 9-0 Monosof (Tyco Healthcare, Wollerau, Switzerland). The lung was first reventilated and then reperfused. A chest tube was inserted and the thoracotomy closed. The chest tube was removed after restoration of spontaneous breathing when the animal was extubated.

#### Intravenous injection of EPC

Immediately before injection of EPC into the host rat, SP-DiIC_18_(3)-labelled cells were harvested, washed and resuspended in EBM-2 medium at a concentration of 1 × 10^6^/ml. Injection of EPC (1 × 10^6 ^cells) was done under general anesthesia with 2% halothane into the saphenous vein of the right hind leg under microscopic vision to ascertain the successful and complete venous administration into each host animal. In preliminary experiments tolerability of intraveinous application of EBM-2 (1 ml) alone turned out to be safe. Intravenous application of EPC was performed 50 to 120 min after reperfusion of the transplanted left lung.

### Assessment of transplanted EPC in the host animal

To evaluate the incorporation of EPC into rat organs, animals were anesthetized by intraperitoneal pentobarbital (50 mg/kg) and ventilated after tracheotomy with an FiO_2 _of 1.0 at 100/min, a tidal volume of 8 ml/kg, and a positive end-expiratory pressure (PEEP) of 5 cm H_2_O. Lung transplanted animals were sacrificed after one day (n = 7), 3 days (n = 1), or 9 days (n = 1) post transplantation. Controls were killed at day one after peripheral EPC injection. Animals were sacrificed after median thoracotomy and intracardiac heparinization with 500 U/kg, when lungs were flushed with 20 ml saline solution through the pulmonary artery. The heart-lung block was excised and the lungs separated: Each lung was divided and one part put into 10% PBS-buffered formalin solution, and the remainder part was deep-frozen in liquid nitrogen and stored at -70°C.

Further organs of the host rats (spleen, liver, kidney and adrenals, stomach, small intestine, colon, bone) were preserved in 10% PBS-buffered formalin solution as well as deep-frozen in liquid nitrogen and stored at -70°C.

### Immunofluorescence staining of tissue specimens

The formalin-fixed tissue was paraffin-embedded and cut at 4 μm to 10 μm (as given in detail in some experiments). Slides were heated in an incubator at 70°C for 30 min before they were deparaffinized in xylene and hydrated in graded ethanol. Slides were incubated with FITC-labelled lectin from *Bandeiraea simplicifolia *(*Griffonia simplicifolia*) BS-I (Sigma, St. Louis, MO) and 3', 6'-diamidino-2-phenylindole, dihydrochloride (DAPI; Molecular Probes, Leyden, The Netherlands) according to the manufacturers' protocol. *Bandeiraea simplicifolia *lectin was chosen due to its affinity to EC, and DAPI staining was used to stain nuclei specifically with blue fluorescence. Lectin was diluted at 1:100 and DAPI at 1:1000 in PBS containing 1% bovine serum albumin (BSA). Analysis was performed by three of the authors (H. N., J.H., C.M.K.) using a Zeiss Axioskop 2 light and fluorescence microscope (Zeiss, Göttingen, Germany). For additional confocal microscopic analysis, histological sections with a thickness up to 10 μm (left-sided injured lung, right lung and the other organs investigated) were examined with an Inverse Axiovert 100 M BP (Base Port) confocal microscope LSM 510 (Zeiss, Göttingen, Germany) using the following laser emissions: DAPI: excitation 364 nm, emission BP 385–470 nm; FITC: excitation 488 nm, emission BP 405–430 nm; SP-DiIC_18_(3): excitation 543 nm: emission LP 585 nm. Fluorescent signals from DAPI, FITC-lectin and SP-DiIC_18_(3) were viewed simultaneously in separate detector channels. True color overlays of single and serial sections were generated with Zeiss confocal software 2.8 SP1.

### Statistical analyses

Values are presented as mean ± S.E.M. The values were compared by Mann-Whitney U test as given in the text. Differences were considered statistically significant at *p *≤ 0.05.

## Results

### Characteristics of ex vivo-generated bone marrow-derived rat EPC

**Figure 1:**
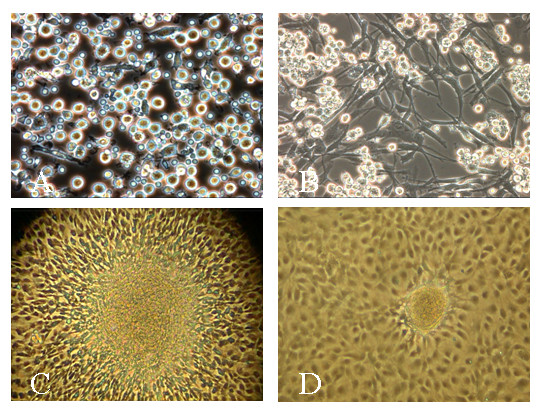
***Rat bone marrow-derived EPC in culture***. A highly purified population of EPC was isolated from hindlimb bone marrow of male Sprague-Dawley rats and maintained in EBM-2 medium containing several growth factors (A). Typical morphology (spindle cell shape) for rat EPC (B) occurred after a few days in culture (phase contrast microscopy 30×). Outgrowth of EPC appeared to occur from an angioblast-like cell as already documented for human EPC (C, 30×). Maintaining of the endothelial colonies in specific growth medium resulted in the proliferation of characteristic endothelial cobblestone colonies (D, 20×).

Cells were harvested from the non-adherent fraction of rat bone marrow mononuclear cells after 48 h of culture on fibronectin-coated culture dishes (Figure 1A). EPC appeared to grow out of a so-called angioblast as had been already described for human EPC [64] (Figure 1C, D). The outgrowth cells first exhibited a spindle cell shape (Figure 1B), and after 7 to 10 days in culture a more endothelial cell-like cobblestone morphology was observed (Figure 1D).

**Figure 2:**
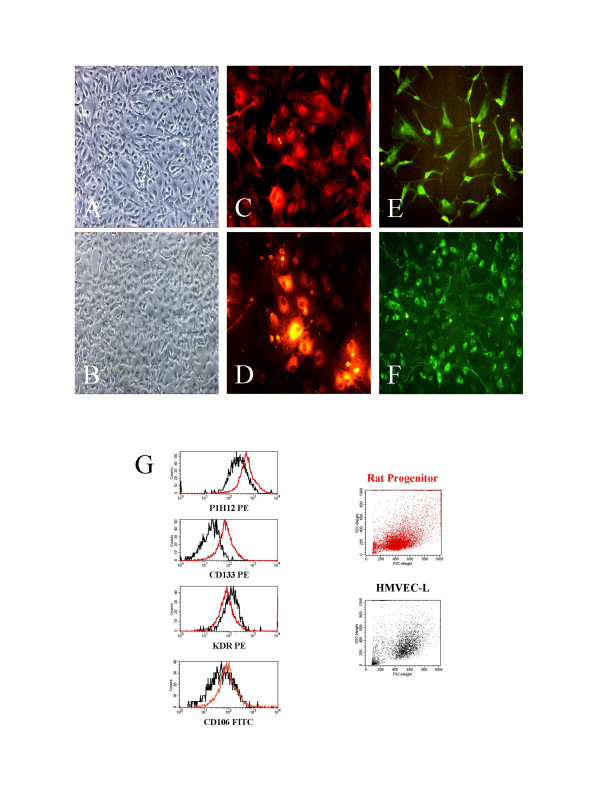
***Characteristics of rat bone marrow-derived EPC***. Utilizing phase contrast microscopy as well EPC (Figure 2A, 20×) as rAEC (Figure 2B, 20×) showed a typical cobblestone morphology after reaching confluence. The endothelial phenotype was further confirmed by immunostaining with antibodies specific for several endothelial markers and compared with mature rAEC: EPC cultured from rat bone-marrow incorporated acetylated low-density lipoprotein (aLDL, Figure 2c, 30×) to the same extent than observed in mature rAEC (Figure 2d, 30×). Furthermore, as well EPC (Figure 2E, 30×) as rAEC (Figure 2F, 20×) uniformly expressed von Willebrand factor (vWF). Figure 2G and 2H show the flow cytometric (FACS) characteristics of EPC (red lines) compared to hL-MVEC (black lines). Staining was performed for P1H12 corresponding to CD146, CD133, the VEGF-receptor 2 (KDR) and CD106 (VCAM-1) as given in Materials and Methods.

Utilizing phase contrast microscopy as well bone marrow-derived EPC (Figure 2A) as rAEC (Figure 2B) showed typical endothelial cobblestone morphology after reaching confluence. The endothelial phenotype was further confirmed by immunostaining with antibodies specific for several endothelial markers and compared with mature rAEC. EPC incorporated Dil-acLDL (Figure 2C) as observed in mature rAEC (Figure 2D). Furthermore, EPC (Figure 2E) as well as rAEC (Figure 2F) uniformly expressed vWF in their cytoplasmic granules. EPC further showed positive staining for CD34, CD31 and VEGF receptor-2 (KDR; Flk-1) in immunofluorescence experiments (data not shown).

Additionally, expression of endothelial surface markers (Figure 2G) on EPC and hL-MVEC were compared by flow cytometry. EC showed a bright staining with CD146-PE (P1H12) and are not stained with the platelet marker CD42d-FITC and the leukocyte marker CD45-FITC. Those antibody combinations ensure that no leukocytes or platelet aggregates with non-specific CD146-PE staining are gated as EC. As depicted, EPC and hL-MVEC express the markers CD146 (P1H12), CD133, KDR and CD106. However, EPC showed higher expression of the stem cell marker CD133 as well as CD146 (P1H12) than mature microvascular endothelial cells.

Appearance of these tested markers was comparable further with staining intensity of mature rAEC. Thus, the expression of diverse EC markers detected confirmed the endothelial identity of the outgrowth cells of the non-adherent cell fraction cultured from rat bone marrow. The endothelial phenotype remained constant for more than 20 passages, demonstrating the stability of freshly isolated EPC from rat bone marrow.

### Proliferative kinetics of rat bone marrow-derived EPC in vitro

**Figure 3:**
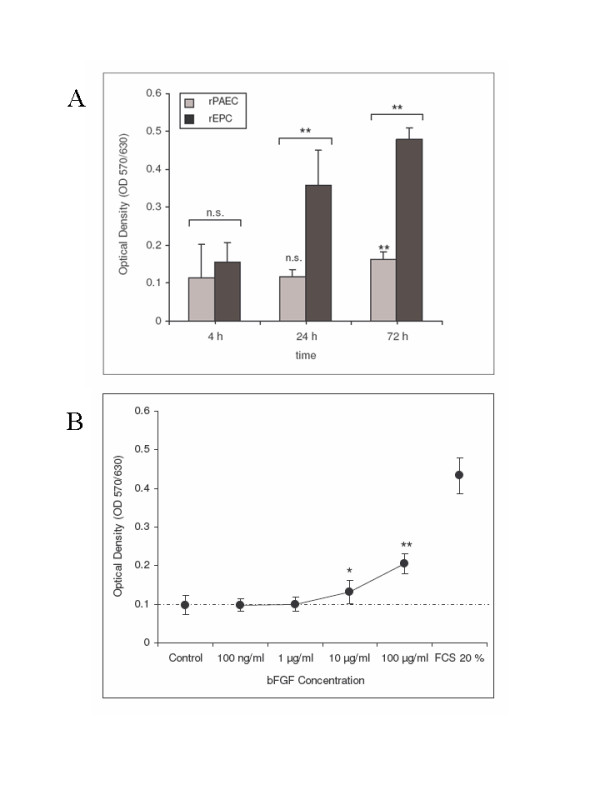
***Proliferative characteristics of bone marrow-derived EPC***. EPC and rPAEC were cultured in the presence of 20% FCS in EBM-2 without further supplements (Figure 3A) or in EBM-2 containing bFGF (Figure 3B). Figure 3A shows the differential growth capacities of EPC and mature rPAEC in the presence of EBM-2 supplemented with 20% FCS. Figure 3B shows the proliferative kinetics of EPC cultured in EBM-2 supplemented with 0.5% FCS towards increasing concentrations of bFGF (100 ng/ml to 100 μg/ml). Cells were seeded in 96-well plates, grown for 24 h in culture medium, washed twice with HEPES/EDTA and treated with bFGF in EBM-2 medium containing 0.5% FCS for 48 h as indicated. Cells were incubated with MTT solution, lysed and the absorbance was read. MTT activity is expressed as Optical Density and represents mean ± SEM of five independent experiments, ** *p *< 0.01, * *p *< 0.05.

As depicted in Figure 3A, EPC show a dramatic increase in their proliferative kinetics when compared with mature rPAEC after stimulation with 20% FCS. The increase in cell number was about threefold when compared with mature rPAEC. These observations are in good agreement with published data by Bompais et al. [45]. Also increasing concentrations of basic fibroblast growth factor (bFGF) resulted in a significant increase of EPC cell number with a maximal effect observed between 10 μg/ml and 100 μg/ml after 72 h (maximal concentration tested; Figure 3B).

### Vasculogenic properties of rat bone marrow-derived EPC

**Figure 4:**
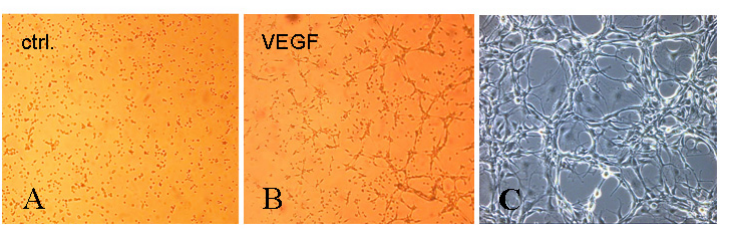
***Capillary-like structures formed by rat bone marrow-derived EPC***. Formation of tubular structures on Matrigel by EPC as a form of organisation characteristic of EC. EPC stimulated with VEGF (50 ng/ml) exhibited more tubular formation and with a particular tendency to multiple links between cell nest (Figure 4B) than observed with the control: hL-MVEC (Figure 4A) However, EPC have the capability to form capillary like structures when seeded at low cell numbers even in the absence of high concentrations of cytokines which suggests their high angiogenic potential. Picture represents a 20× magnification phase contrast microscopic picture (Figure 4C).

Consistent with the observed endothelial phenotype (phase contrast microscopy, detection of endothelial markers by flow cytometry), EPC formed capillary-like formations within 6–12 hours when plated on Matrigel after stimulation with VEGF (50 ng/ml; Figure 4B) when compared with the angiogenic potential of hL-MVEC (Figure 4A). Even spontaneous formations of capillary-like structures were observed by EPC when seeded at low cell numbers on fibronectin-coated (10 μg/ml) cell culture plates (Figure 4C). However, after cell number of EPC increased they showed a more cobblestone morphology (Figure 1, 2). These observations suggest a high capacity of EPC to form new vessels.

**Figure 5:**
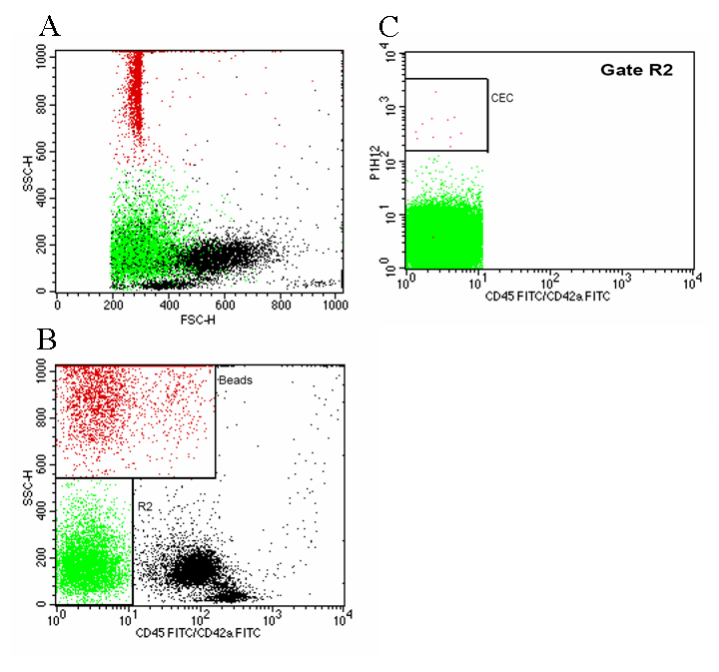
***Flow cytometry analysis of circulating EC after transplantation***. Flow Cytometry Analysis of Circulating EC after Transplantation. Example of a flow cytometric analysis of circulating EC in peripheral blood. The depicted data show a blood sample from an EPC-injected lung transplant recipient 12 h post reperfusion. Whole blood was stained with CD42d-FITC to exclude platelet aggregates and with CD45-FITC to exclude leukocytes, as well as CD146-PE (P1H12) as second fluorescence. Circulating EC are defined as CD146 positive and negative for CD45 and CD42d. The localization of that cell group in forward and side scatter is shown in Panel A as the green cell group. Panel A and B contain furthermore as a red group a standard dose of control beads in order to quantify cells. Panel A shows the forward and side scatter characteristics of the blood sample. In panel B the latex beads which are used as internal quantification standard (gate Beads) and the CD45 and CD42a negative population (gate R2) are gated. In panel C the CD45 and CD42a negative population of R2 is analyzed for their expression of the endothelial cell marker P1H12. Cells were counted as circulating endothelial cells when they were included in the depicted gate CEC.

### Flow cytometry analyses of circulating EC after peripheral EPC transplantation

Flow cytometry analyses with species cross-reactive monoclonal antibodies showed baseline levels of 302 (SD 222) circulating EC/ml peripheral blood in untreated animals. 12 h after reperfusion the amount of circulating EC (presumably mainly including transplanted EPC) increased to 22.300 (SD 14.190) circulating EC/ml peripheral blood. An example of the flow cytometric measurement and the gating is depicted in Figure 5. This preliminary finding suggests that a rather high number of circulating EC can be detected in the circulation already few hours after initiation of ALI. However, as a limitation of these observations, transplanted EPC could not be determined directly by this method since the erythrocyte lysis buffer, which must be used for the flow cytometric analysis of blood samples, removed the fluorescence of the tracer dye SP-DilC_18_.

### Incorporation of rat bone marrow-derived EPC into the injured left-sided transplanted lung and in subcutaneously administered Matrigel

**Figure 6:**
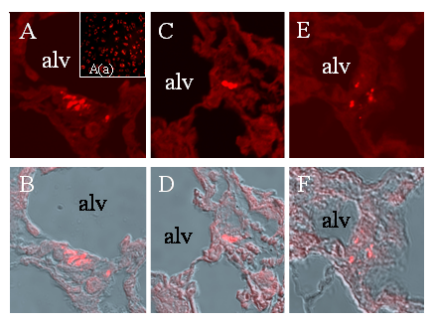
***Localisation of EPC in the left-side transplanted lung***. Lung specimens from rats after left-sided lung transplantation. EPC were stained prior to transplantation with SP-DiIC_18_(3) (red). Figure 6A shows SP-DiIC_18_(3)-staining (red) of EPC prior to intravenous transplantation. A-C, C-D and D-F show the same specimens, respectively. In the upper line confocal fluorescence microscopy for SP-DiIC_18_(3)-stained EPC (red) is showed (A-C). In the lower row fluorescence is overlaid with light microscopy (D-F) for the same specimens. Abbreviation used: alv stands for alveoli.

**Figure 7:**
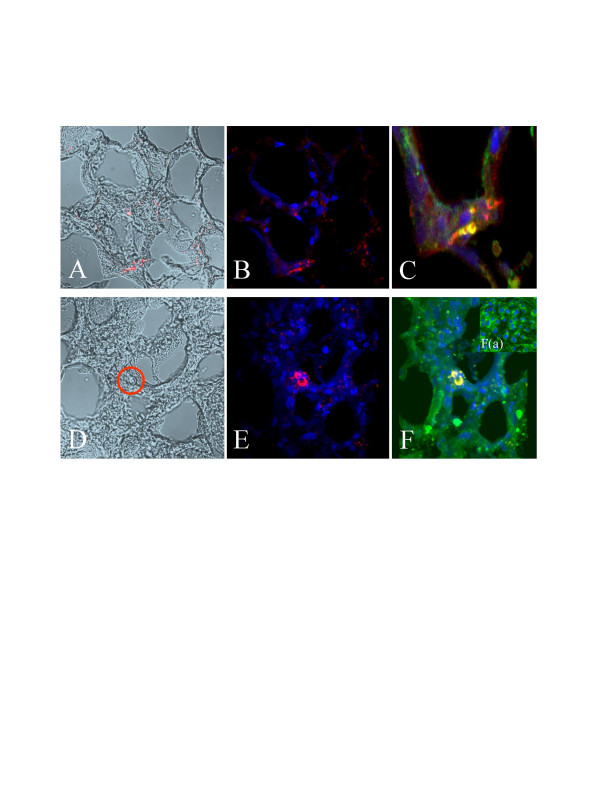
***Localisation of EPC in the left-sided transplanted lung***. All pictures show the same lung specimens from rats after left-sided lung transplantation and EPC injection. EPC were stained prior to transplantation with SP-DiIC_18_(3) (red), followed by staining of histological specimens with the nuclear dye DAPI (blue) and the more endothelial specific FITC-labeled lectin from *Bandeiraea simplicifolia *(*Griffonia simplicifolia*) BS-I (green) showing the distribution of EPC. A-C: A-SP-DiIC_18_(3) (red), B – SP-DiIC_18_(3) (red) and DAPI (blue), C – merge: SP-DiIC_18_(3) (red), DAPI (blue) and FITC-labeled lectin from *Bandeiraea simplicifolia *(*Griffonia simplicifolia*) BS-I (green) – Orange reveals staining for red and green) [Figure 7C represent magnification and a merge picture of a marked section of Figure 7A, B]; D-F: D: SP-DiIC_18_(3) (red), E: SP-DiIC_18_(3) (red) and DAPI (blue), F – merge: SP-DiIC_18_(3) (red), DAPI (blue) and FITC-labeled lectin from *Bandeiraea simplicifolia *(*Griffonia simplicifolia*) BS-I (green) – Orange reveals staining for red and green). F(a): Staining of EPC in culture with DAPI (blue) and FITC-labeled lectin from *Bandeiraea simplicifolia *(*Griffonia simplicifolia*) BS-I (green) Abbreviation used: alv stands for alveoli.

SP-DiIC_18_(3)-labeled EPC were administered intravenously 50 to 120 min after reperfusion of the transplanted left sided lung after a cold ischemia for 20 h. This unilateral orthotopic lung transplant model leads to a severe ischemia-reperfusion injury resulting in an ALI in the transplanted lung leading to a P_a_O_2_/F_i_O_2 _of about 50 – 70 mm Hg [63]. Already one day after transplantation, SP-DiIC_18_(3) – labeled EPC were detectable in the injured lung tissue. Specific immunofluorecence for SP-DiIC_18_(3), was found throughout the left lung in all left lung transplanted animals (n = 9) while in the right lung or other organs of the same rats transplanted EPC were virtually not found. In all lung sections investigated we were able to detect injected EPC at a number of about 10 to 15 cells per slide. As a rat lung is about 30.000 μm long we can suggest that about 30.000 (3%) up to 112.500 (11%) of EPC transplanted may home in the injured left lung (Figure 6). However, as a limitation of the method used (in vivo cell labeling) staining of partner cells upon cell fusion can not be excluded completely. *Ex vivo *generated EPC seemed to be incorporated into pulmonary capillaries as suggested by double-staining with lectin BS-I and nuclear staining with DAPI (Figure 7). Still 9 days after injection of SP-DiIC_18_(3)-labeled cells, EPC were detectable in the transplanted left lung and seemingly integrated in capillary-like structures therein but not in the non-transplanted right lung. EPC were attributed to alveolar septal capillaries while we observed only few immunofluorescent signals in larger pulmonary vessels. Furthermore, SP-DiIC_18_(3)-labeled EPC were also detected within widened septa of thickened alveoli. These cells could not be directly attributed to patent vessels. Interestingly, no EPC were found in alveolar spaces or in vessel lumina ever. DAPI staining confirmed functional integrity of injected EPC (Figure 7). In control animals (n = 2) virtually no peripheral administered EPC could be found in lung tissue. However, only about 0.5% of DAPI positive cells were co-labeled SP-DiIC_18_. These observations are in good agreement with recently published data concerning peripheral injection of GFP-expressing EPC in immunodeficient (F344/N rnu/rnu) nude rats [65].

**Figure 8:**
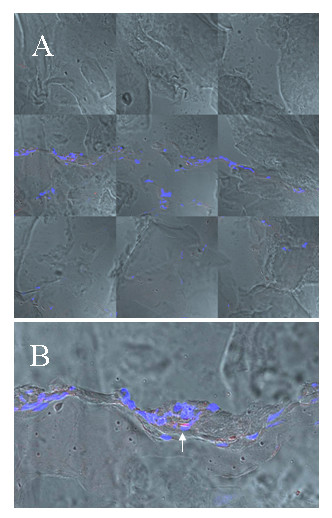
***Localisation of EC and EPC in subcutanous matrigel***. Confocal fluorescence microscopic examination of a subcutanously administered Matrigel, an extracellular mouse sarcoma matrix (Engelbreth-Holm-Swarm tumor) known to be *in vivo *a pro-angiogenic stimulus, that had been administered to the rats with EPC injection one week before transplantation subcutanously. Matrigel showed invasion of cells building up capillary like structures. EPC are positive for SP-DiIC_18_(3) and DAPI. Figure A,B; blue: DAPI, red: SP-DiIC_18_(3)-labeled EPC).

EPC were not detectable in the investigated specimens of other tissues such as myocardium, kidney and liver by fluorescence microscopy so far. However, EPC have been found in subcutaneously administered Matrigel which had been administered 8 days before lung transplantation in six animals. Figure 5 confirms both, that vessels have sprouted into the matrix and that about 24 h after left sided lung transplantation and about 23 h after EPC administration some of the peripherally injected EPC have invaded the Matrigel as evidenced by confocal microscopy (Figure 8). 

## Discussion

The main finding of this study of one-sided severe ALI by ischemia and reperfusion is that incorporation of EPC could be demonstrated in the injured lung vascular bed and within the damaged tissue after peripheral administration. EPC were detected at a percentage between 3 to 11% in the left lung in our model. Homing of *ex vivo *generated EPC was selectively found in the injured transplanted left-sided, but not in the right lung (not transplanted). Also other organs like liver, spleen, kidney, stomach or intestines showed no detectable homing, whereas subcutaneously administered Matrigel gave evidence of few cells having migrated in. However, the number of EPC detected in the injured left lung and in the administered Matrigel might be underestimated as after cell division the fluorescent cell marker has been shown to loose its intensity. These findings, together with that of high amounts of circulating EC found after injection of EPC in venous blood, also corroborated that EPC found in the transplanted lung are not explained by simple embolism and suggests that a tropism of such cells to vasculogenic or wound healing areas might occur.

Homing of EPC in injured lung tissue gives evidence of a potential repair mechanism not yet observed in ALI. Indeed, not only the capillary leak that underlines the altered EC filter function of pulmonary microvessels, but also cell death has been described to be a clear feature of such transmural lung injury [5,7]. A high number of capillaries may be destroyed, and EC may undergo apoptosis or necrosis. It is therefore conceivable that EPC home at these pulmonary vascular sites where the adult EC phenotype is demised. This hypothesis is supported further by a recent study of Nagaya *et al*. [65]. They showed in a rat model that homing of EPC in pulmonary hypertension occurs and ameliorates monocrotaline-induced pulmonary hypertension, similar to recent work of Zaho *et al*. [66], suggesting that apoptotic mature EC are replaced by novel functional cells. Further work from Davie *et al*. gave evidence of a participation of EPC in adventitial vasa vasorum in a hypoxia model [67]. A number of similar approaches have been described. Mouse studies on hindlimb ischemia have shown an enhanced tissue neovascularization with increased blood flow and capillary density [68] and a significantly lower number of lost limbs due to the transplantation of human EPC in athymic nude mice [69]. A pilot study and randomized controlled trial in limb ischemia patients treated with autologous transplanted bone-marrow cells in ischemic limb muscles showed a sustained significant effect of such therapeutic angiogenesis [35].

Thereby, it is noteworthy that cell demise seems a critical phenomenon in ALI. Apoptosis seems to be a major pathway to EC death in ALI, and by the use of a caspase inhibitor to block the execution of apoptosis, there is some indirect evidence that influencing such apoptosis may affect animal survival [9]. On the other hand, apoptosis may be under circumstances of infection protective for the animal survival [70]. In our utilized lung injury model such apoptosis has been demonstrated to occur [71]. Second, it gives first evidence that such a stem cell-based replacement of dying or dead cells in the lung may be accomplished as a therapeutic strategy by an intravenous cell transplantation approach in severe ALI [72].

Furthermore, a CD34-negative subpopulation of bone marrow cells has been shown to uniquely engraft and reconstitute a minute part of ischemic myocardium with cardiomyocytes and EC [73]. The very similar finding of EC engraftment contrasts with the quite dissimilar cell population they used. Whether the difference in used populations may be of less importance due to the plasticity of such stem cells or progenitor cells [74,75] that may be able to transdifferentiate or dedifferentiate and even cross germ lines, or due to the difficulty to define such cell populations [76] remains open. Also cell fusion [77-79] might be a reason for such a trans-or dedifferentiation hypothesis and may further increase the difficulty to categorize such cells. Observed controversies might be part of the different origins of EPC used in these studies [80].

Recently, Voswinckel *et al*. investigated after reporter gene bone marrow grafting in a model of left-sided pneumonectomy the compensatory lung growth that leads to important alveolization in rodents [81]. They could not find bone marrow-derived EC or smooth muscle cells, pericytes or fibroblasts in their model of rather slow regeneration and alveolarization where no injured lung tissue is present. Their very thorough approach to use three different mouse strains may imply, contrary to our finding in an ALI model, that the proliferative capacity of endogenous cell compartments of the lung [11] would be sufficient for such regeneration in their model of rather slow regeneration.

On the other hand it has been suggested that circulating bone marrow-derived stem cells support tissue-specific cells during periods of severe acute injury in different tissues [82-84], or even repair more generally. A recent study by Yamada *et al*. corroborated the cell substitution hypothesis in the lung after pulmonary LPS exposure in mice. They observed a rapid mobilization in terms of an increase of bone marrow-derived progenitor cells in the circulation 4 h after exposure by about a factor of four, an accumulation of those cells within inflammatory sites and then their differentiation to endothelial or epithelial cells [13]. If progenitors were suppressed by body irradiation, within one week the mice developed emphysema-like lesions, probably due to missing substitution of apoptotic or necrotic cells [10] and similar to an emphysema model of repeated LPS exposure [85]. In contrast, mice with LPS exposure and intact bone marrow did not have such structural changes one week later. These findings suggest that an inflammatory stimulus does not only induce the release of inflammatory cells from the bone marrow, but also that of progenitor cells. Furthermore, these cells might be crucial to repair the lung in order to maintain the organ structure, as they integrate in the tissue and seem to differentiate or to fuse with other parenchymal cells to endothelial or epithelial cells. Whether these progenitors may have a more general therapeutic role in inflammatory diseases to repair lung parenchyma or even in diseases with important chronic lung destruction like emphysema remains open [13].

A number of studies addressed the role of such circulating progenitors: their mobilization [86,87], homing [88] and their association with inflammation [43,89], pneumonia [56], pulmonary hypertension [65-67], acute lung injury [55], or cancer [57,90,91]. Furthermore, we were able to detect CD133-positive EPC in tumor tissue of patients suffering from bronchial carcinoma [92], a concept that has been questioned experimentally [93]. Further studies are necessary to better understand their dynamics in such a repair process in health as well as in the addressed disease states.

As a limitation of our study protocol we can not give evidence of functional improvement by EPC homing. However, there are other reports from studies on therapeutic strategies that such transplantation of EPC may be favorable. Indeed, injured arteries or bio-prosthetic grafts have been shown to be early re-endothelialized with administered EPC, apparently resulting in less neointima deposition [46,94]. The progressive loss over time of transplanted cells in the grafts might be a result of rapid cell turnover and simultaneous replacement by recipient cells, be it from bone marrow or from nearby. Whereas similar findings were also made with another study using transfected cells [95], a definitive answer on the rapidity of cell replacement is still lacking due to technical limitations of such studies.

## Conclusion

We could demonstrate the integration of intravenously injected *ex-vivo *expanded bone marrow-derived EPC in the injured vasculature uniquely of the transplanted lung with ALI. Those transplanted EPC may play a central role in reestablishing the endothelial integrity in injured vessels [13] and might further contribute in wound healing processes [21,55]. Our observations may have implications for novel cell-based therapeutic strategies [96], be it as endothelia per se to establish the vascular integrity, be it as vector for mediators as already shown with bone-marrow derived cells for the pulmonary epithelium [97] or as a "Trojan horse" aiming at tumor vessels for therapeutic interventions.

## Competing interests

The author(s) declare that they have no competing interests.

## Authors' contributions

C.M Kaehler made substantial contribution to the conception, design, aqcuisition of data, analysis of data and interpretation thereof; J. Wechselberger performed charcterisation and isolation of EPC; W. Hilbe was engaged in evaluation of immunoimmunochemistry and fluorescence microscopy; D. Colleselli did the proliferation experiments; H. Niederegger performed the confocal florescent micropscopy; E. Boneberg did all FACS experiments; G. Spizzo was associated with the preperation of pathological specimens; A. Wensel, E. Gunsilius and J.R. Patsch have been involved in given final approval of the version to be published. J Hamacher performed all experimental animal procedures, the vasculogenesis experiments in vitro and made substantial contribution to the conception, design, aqcuisition of data, analysis of data and interpretation thereof. All authors read and approved the final manuscript.
